# Residue-Resolved Liquid-State Hyperpolarized NMR of Peptide Condensate Surfaces

**DOI:** 10.1021/jacs.5c15053

**Published:** 2025-09-25

**Authors:** Dörte Brandis, Ertan Turhan, Milan Zachrdla, Dennis Kurzbach

**Affiliations:** † Institute of Biological Chemistry, Faculty of Chemistry, 27258 University of Vienna, Währinger Str. 38, 1090 Vienna, Austria; ‡ Vienna Doctoral School in Chemistry (DoSChem), 27258 University of Vienna, Währinger Str. 42, 1090 Vienna, Austria

## Abstract

Understanding how biomolecular condensates interact with their environment requires atomic-level insights into their surface composition. However, conventional Nuclear Magnetic Resonance (NMR) spectroscopy lacks the sensitivity to probe solvent-exposed regions in large, phase-separated peptide systems, where surface moieties are sparse relative to bulk residues. Here, we introduce hyperpolarized liquid-state surface-specific NMR spectroscopy, a technique that visualizes solvent-accessible residues in large biomolecular condensates. In combination with unconventional sample handling instrumentation and hyperpolarization-specific data processing, we report high-resolution hyperpolarized surface NMR spectra with spectral qualities that do not fall short of cutting-edge high-field methods yet have substantially boosted sensitivity. Targeting the biotechnologically widely used elastin-like polypeptide (ELP) nanoscale complexes, we demonstrate residue-resolved detection of the water interface of mega Dalton-sized peptide condensates with sensitivity enhancements reaching 2 orders of magnitude. Our method reveals glycine residues at the coacervate surface while hydrophobic core residues remain suppressed, providing direct evidence for glycine-rich surface segregation in these biomaterials. These findings resolve long-standing questions about ELP surface architecture and open an avenue for a solution-state analog to surface-enhanced solid-state NMR. The presented advance might thus foster detailed investigations of soft-matter interfaces in protein condensates, synthetic coacervates, and bioengineered materials.

## Graphical abstract

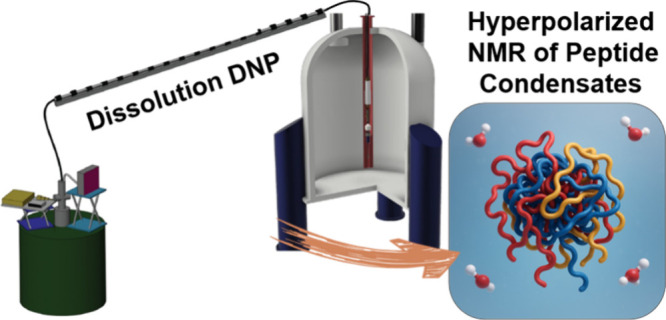

## Introduction

Nuclear Magnetic Resonance (NMR) spectroscopy is unparalleled in its ability to resolve the structural and dynamic properties of biomolecules at atomic resolution in solution.^1,2^ However, its full potential remains curbed by the inherently low sensitivity of the technique.^3^ This limitation becomes especially critical for low-abundance moieties at the solvent interfaces of large biomolecular condensates consisting of many assembled proteins or peptides. The small number of surface residues relative to the bulk leads to weak signals that often approach or fall below detection thresholds.^4–7^

This is a critical flaw as surface regions or solvent interfaces of large complexes, coacervates, or self-assemblies are often crucial for biomolecular activity.^8–10^ They govern the physicochemical interactions with the environment and always represent the first point of contact with any interactant or ligand.^11–13^ They are, thus, key determinants of function in biomaterials, drug delivery systems, and intracellular phase transitions. At the same time, these interfacial regions regulate access to vital buried domains, such as hydrophobic cores, which often play essential roles in catalysis, stability, and allostery.

To address the NMR-sensitivity bottleneck, recent developments in dissolution dynamic nuclear polarization (dDNP) have pushed applications for signal enhancements in biomolecular applications.^14–16^ Among them, hyperpolarized water (HyperW) has emerged as a powerful tool for boosting polarization, i.e., signal intensity of labile, solvent-exposed protons in biomolecular targets.^17–34^ In this method, HyperW is generated ex situ in a cryogenic DNP system and transferred to an NMR spectrometer, where it is mixed with a target molecule. Polarization is conveyed to labile sites of the biomolecule primarily via proton exchange (and to some extent NOE). In this way, HyperW-based NMR renders previously undetectable species observable and opens access to conformational states and interaction sites that are inaccessible with conventional NMR sensitivity.^17–34^

While prior studies have focused on the application of HyperW for monitoring protein folding,^30^ ligand binding,^17^ or molecular interactions,^24,29^ its capability to resolve large peptide condensates, and even liquid–liquid phase separation^35–37^ remains unexplored. Yet, such targets represent one of the most critical frontiers in current structural biology, and significant efforts are made to access them, from advanced NMR methods such as methyl-TROSY,^38–42^ to solid-state techniques^43–46^ and the use of artificial intelligence.^47–49^

Despite these ongoing efforts, NMR methodologies still often fail to access the surface of biomolecular condensates, consisting of many copies of the same peptide or protein, with sufficient sensitivity. This limitation arises as the number of solvent-exposed residues at the particle interface is minimal compared to the total condensate content, resulting in extremely low signal intensities relative to the bulk of resonances.^50,51^

In this study, we overcome this hurdle by employing HyperW in combination with tailored DNP ↔ NMR interface instrumentation and data processing. Thus, we achieve high-resolution spectra composed of selectively boosted NMR signals of surface moieties in mega Dalton-sized peptide condensate reminiscent of a liquid–liquid phase-separated peptide droplet. HyperW transfers magnetization to labile hydrogens via proton exchange, thereby selectively enhancing signals from solvent-exposed moieties while suppressing contributions from structurally shielded sites within the assemblies’ cores (Figure 1).

**Figure 1:**
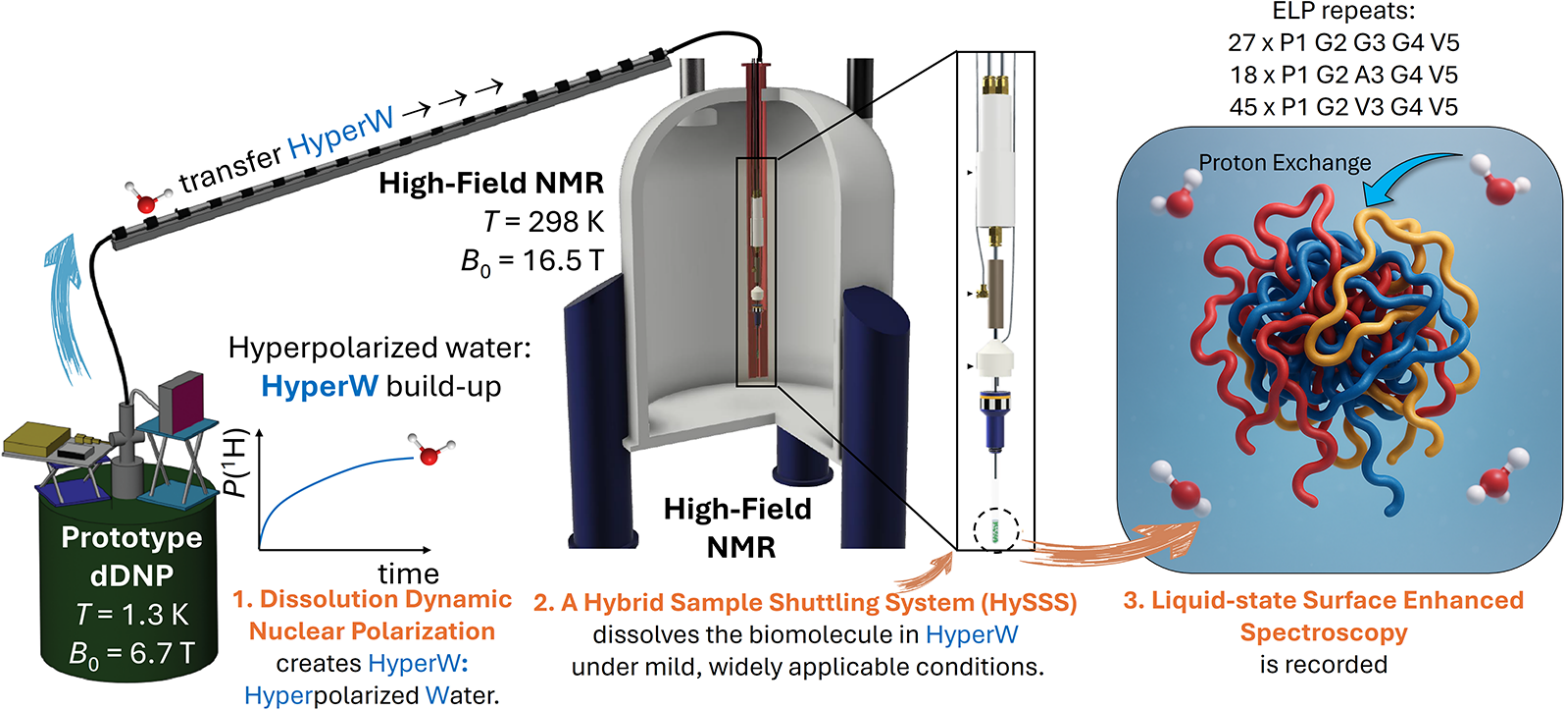
Experimental concept. (1) Water is hyperpolarized, i.e., signal boosted at cryogenic temperatures. Upon completion of the build-up, the water pellet is dissolved and transferred to an NMR spectrometer for detection. (2) Custom hybrid sample shuttling system (HySSS.v2), inserted directly into the bore of the NMR spectrometer, collects the HyperW (the HySSS.v2 is explained in detail in the Supporting Information). This device mixes the HyperW under mild, controlled conditions with the target system. Here, a mega Dalton-sized self-assembled ELP complex (peptide sequence indicated and detailed in the Supporting Information). Subsequently, it forwards the sample to the NMR tube. (3) ELP self-assembly (simplified sketch) exchanges solvent-exposed protons with the hyperpolarized water, transferring signal intensity from the buffer to its surface to allow for the enhanced NMR detection.

We focus on the surfaces of nanoscale complexes formed through self-assembly of elastin-like polypeptides (ELP).^52–54^ ELPs are an important platform for current molecular medicine and drug delivery developments.^52,55–57^ They self-assemble above a tunable lowest critical solution temperature (LCST), thereby unfolding their guest-molecule-hosting capacities for controlled transport and release. Despite enormous efforts to understand the conformations of these self-assemblies,^58–65^ the solvent-ELP assembly interfaces remain entirely unknown. Such knowledge is key when rationally designing ELPs for tailored functionality.

Herein, with the signal boost by HyperW, we atomistically resolve ELP-solvent interfaces. By exploiting the selective sensitivity enhancement provided by HyperW, we characterize the interfacial residues of ELP coacervates, providing atomistic insights into their surface topography. This development offers a new spectroscopic handle on the interface chemistry of coacervates, biomolecular condensates, and related nanostructures, all within their native solution environment.

## Results and Discussion

To enable liquid-state surface spectroscopy of coacervated biomolecular assemblies, we established a protocol integrating HyperW with a newly customized hybrid sampling shuttling system (HySSS.v2) prototype, which enables mixing of HyperW with a coacervate suspension under mild, pressure- and temperature-controlled conditions that do not perturb the solution-state equilibrium (such as earlier implementation detailed in ref 66. HySSS.v2 contrasts conventional dDNP setups, where the hyperpolarized solutions are often mixed with other solutions under high-pressure and often also high-temperature conditions. Thus, it renders dDNP investigations of ELP condensates possible.

The core concept of the experimental design, including the HySSS.v2 as an interface between DNP and NMR systems, is outlined in Figure 1. Details can be found in the Supporting Information (Figures S4–S6).

Furthermore, we have introduced a special apodization function that takes into account the decay of the hyperpolarized water during data acquisition. While this decay typically introduces distortions in HyperW experiments, correcting for it during data apodization recovers native line shapes. All details are laid out in the Supporting Information (Figure S7). In combination with the HySSS.v2, we could thus record hyperpolarized data with similar line shapes as found in conventional high-field NMR: gentle mixing by HySSS.v2 preserves sample homogeneity; tailored apodization cancels HyperW-decay-induced distortion.

With the setup of Figure 1, proton-hyperpolarized water is generated ex situ via cryogenic DNP (*T*_DNP_ = 1.4 K; *B*_0,DNP_ = 6.7 T). The hyperpolarized solvent is then rapidly transferred into a liquid-state NMR spectrometer and mixed by the HySSS.v2 with a solution or emulsion of the target system (*T*_NMR_ = 298–333 K; *B*_0,NMR_ = 16.5 T). The waiting sample is composed of dispersed ELP (below the LCST) or the equivalent coacervated droplets (above the LCST) suspended in an aqueous buffer.

Herein, the ELP not only serves as a prototypical model for large assemblies of intrinsically disordered proteins but also displays a system of significant, timely biomedicinal interest.^52,56,57,67–70^ Thus, to render our study as representative as possible, we employed one of the most widely used ELP^71^ in actual applications, a 450-amino-acid-long variant with 27 G, 18 A, and 45 V residues randomly distributed as the X in a total of 90 PGXGV pentapeptide repeat (Figure 1). The LCST under our experimental conditions was ∼ 34 °C (see the Supporting Information, Figure S1).^72^ At this temperature, the ELP forms assemblies of ca. 150 nm in diameter (as shown by DLS Figure S2), consisting of hundreds of peptides assembled in one structure.^72^ The cumulative molecular weight, thus, significantly exceeds 1 MDa (see the Supporting Information for a molecular weight estimation).

Figure 2 displays the key problem for NMR detection resulting from the self-assembly. Standard NMR methods, such as the ^1^H–^15^N HSQC, cannot record the peptide complexes. Our earlier work^72^ showed that this is a problem of accelerated chemical and exchange processes within the ELP assemblies, broadening resonances beyond the detection threshold. Indeed, the internal dynamics are reminiscent of a liquid–liquid phase-separated condensate, with moderately reduced dynamics. Thus, slow tumbling only plays a minor role in line broadening. Hence, when surpassing the LCST, most of the signal intensity is lost, and NMR picks up resonances only from residual monomers in solution.^72^

**Figure 2:**
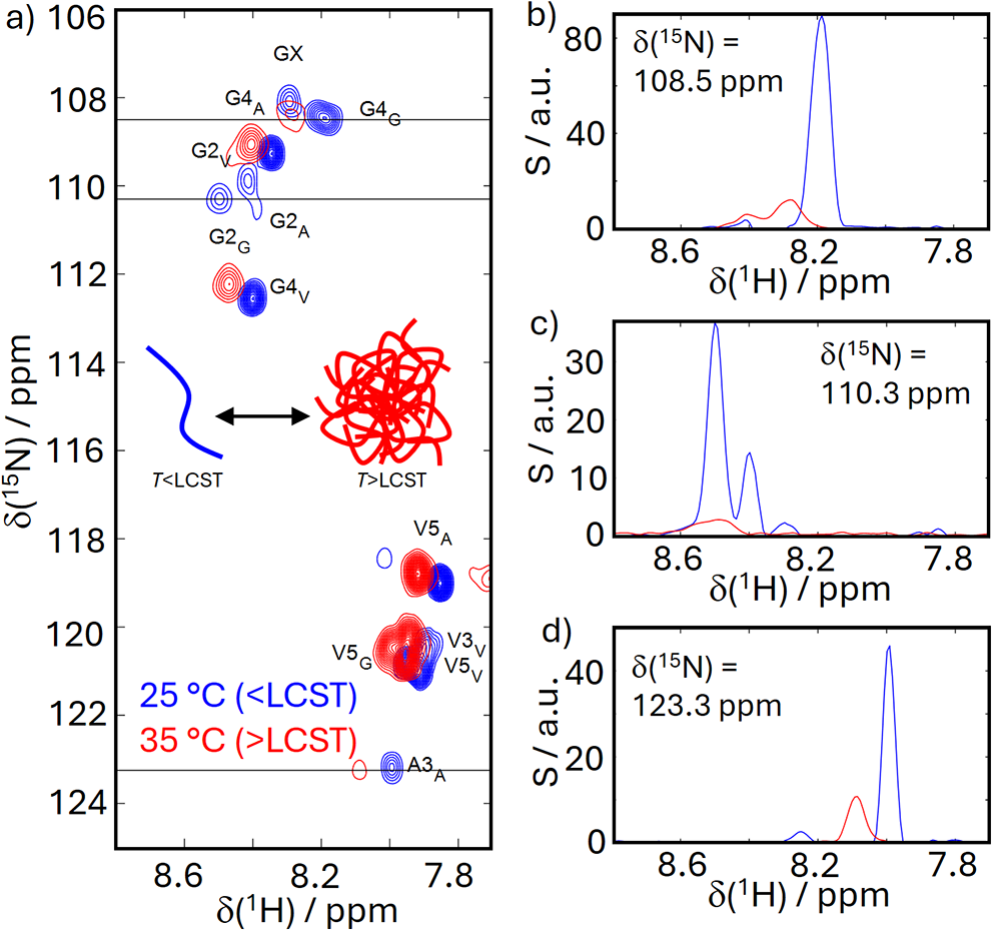
(a) Conventional NMR reference ^1^H–^15^N spectra obtained under conditions optimized for ^1^H^N^ amide-detected NMR in thermal equilibrium (buffer: 90% H_2_O, 10% D_2_O, pH 6.5, and [ELP] = 2 mg/mL) below the LCST (blue) and above the LCST (red). Nomenclature: Residues are indicated with their position in the P1-G2-X-G4-V5 pentapeptide repeats, whose guest residue type X is indicated as subscripts. The entire peptide sequence can be found in the Supporting Information. (b–d) Slices through the 2D spectra (indicated by the black lines) highlighting the signal loss upon aggregation. The residual signals display sharp lines indicative of residual monomers remaining in solution above the LCST.

Our earlier work further showed that the internalized ELP strands remain relatively mobile, reminiscent of liquid–liquid phase-separated droplets, which allowed for their detection by tailored ^13^C-direct detection methods.^72^ However, this approach introduces a different issue: it captures the entire coacervate phase, including its dense core. As a result, the subtle and dilute contributions from surface regions are buried below the dominant bulk signals.

Indeed, for the presented case, it is crucial to note that the repetitive ELP sequence results in a situation where bulk resonances in an assembly of several hundred ELP strands are formed by several hundred similar residues, leading to constructively superimposed signals. In contrast, surface resonances are constituted by only a minute fraction of residues, given the large size of the coacervates.

Hence, the interfacial residues comprise only a small fraction of the total coacervate material and their signals remain weaker by orders of magnitude. The way the ELP stabilizes the water interface of its coacervates, thus, remained unclear. Indeed, conventional NMR strategies are fundamentally ill-suited for resolving molecular details at such coacervate surfaces. The lack of signal strength for resonance read-out similarly hampers all the established means of water-interface detection, such as looped PROSY,^73^ water-selective NOESY,^21^ and many more.^4–7^

What is required is a method that selectively amplifies signals from solvent-accessible regions while preserving spectral resolution; a solution offered by surface-selective HyperW-enhanced NMR, boosting sensitivity by orders of magnitude relative to conventional methods.

As magnetization is enhanced via proton exchange to solvent-exposed amide groups exclusively at the particle–solvent interface, the resulting spectra are dominated by signals arising from the surfaces of the coacervates, allowing for their direct spectroscopic interrogation with residue-level resolution. Such selectivity has been repeatedly validated in recent literature for small globular and intrinsically disordered proteins, where HyperW has emerged as a tool to probe solvent-accessible moieties.^19–21,26,33^

To exploit this effect in the study of large complexes, the experiment outlined in Figure 1 was applied at two representative temperature regimes below and above the LCST (25 and 35 °C, respectively), aiming to delineate the structural reorganization occurring during phase separation (Figure 3). Under these conditions, the HyperW lifetime was ca. 1 min (see the Supporting Information, Figure S3), such that ^1^H–^15^N BEST-HMQC^74^ spectra could be recorded with 256 points (*t*_1_ increments) at a recovery delay *d*1 of 300 ms between each scan (details in the Experimental Section).

**Figure 3:**
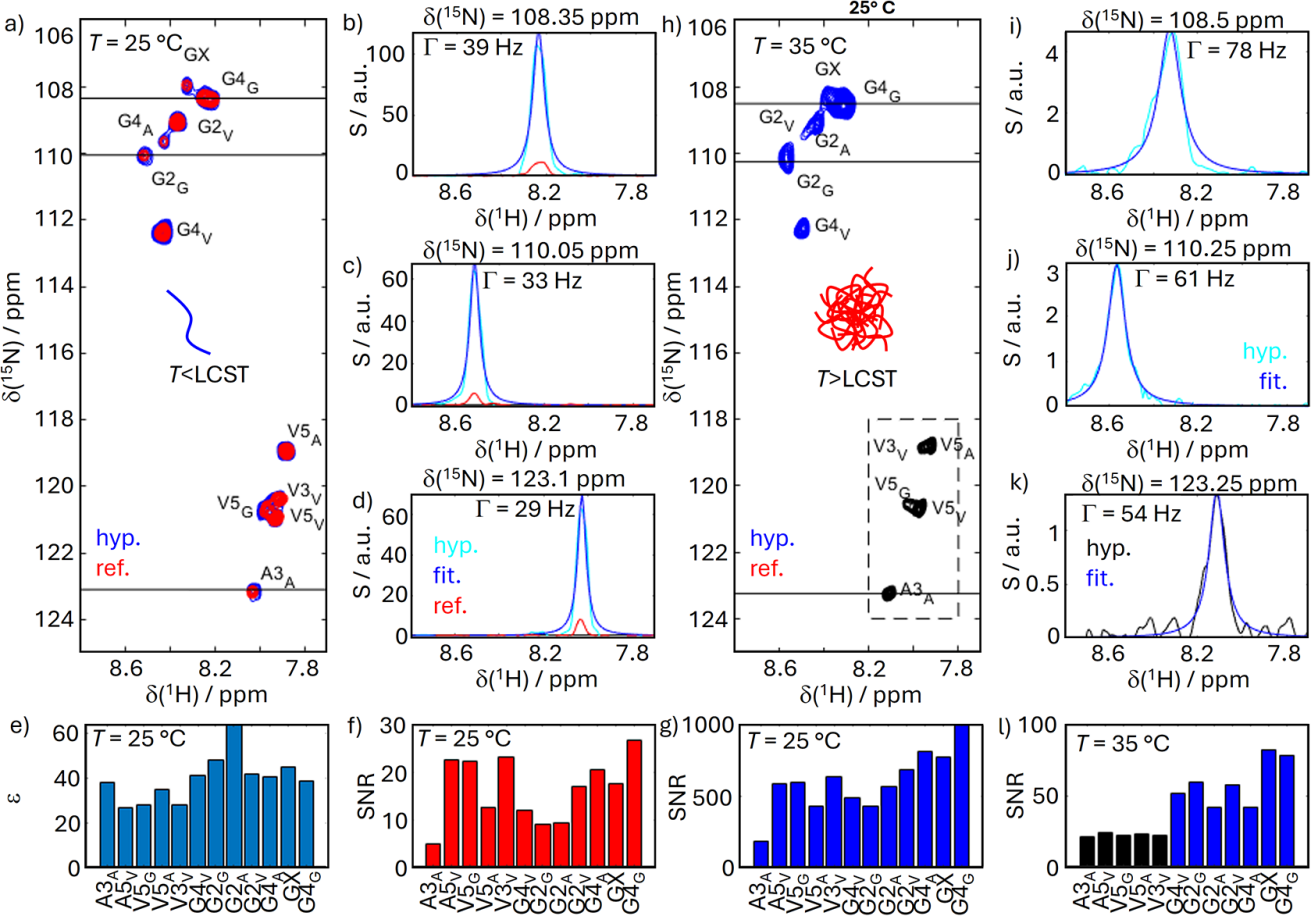
(a) Hyperpolarized ^1^H–^15^N spectra obtained under dDNP optimized conditions (HyperW buffer: 4% HyperW, 96% D_2_O, pH 7, and [ELP] = 0.6 mg/mL) below the LCST (blue) compared to a reference obtained with the same sample and experiment after decay of the hyperpolarization (red). (b–d) Slices through the 2D spectra indicated by the black lines in panel (a). Hyperpolarized spectrum was fit to a Lorentzian function to determine the line width as indicated in each panel. (e) Residue-resolved signal enhancements below the LCST. (f and g) Residue-resolved signal-to-noise ratios (SNR) below the LCST for the reference (red) hyperpolarized (blue) cases. (h) Hyperpolarized ^1^H–^15^N spectrum above the LCST. No reference could be obtained under these conditions. For clarity, black signals within the dashed box are plotted with a different contour cutoff (1.5 times the noise level) than the blue signals (3 times the noise level). (i–k) Slices through the spectrum in panel (h) as indicated by the black lines and fits of the peaks to Lorentzian functions to obtain the indicated line shapes. (l) SNR values for the hyperpolarized spectrum above the LCST. The color code matches the spectrum in panel (h).

### Below the LCST

The polypeptides remain fully solvated in a homogeneous solution. Upon injection of hyperpolarized water, polarization is transferred uniformly to solvent-exposed amide groups along the ELP chain. The resulting hyperpolarized spectra showed several key features.

First, Well-resolved signals emerge in the hyperpolarized spectra, corresponding to the dispersed and dynamically mobile state of the polypeptides. These spectra display contributions from all residues, reflecting their equal exposure to the aqueous phase (Figure 3a).

Importantly, the line widths (Figure 3b–d) in the hyperpolarized spectra were consistent with monomer peptides (between 29 and 39 Hz as determined by Lorentzian fits) and resemble those found in the conventional NMR spectra in Figure 2. The line width also did not change in reference spectra obtained after decay of the hyperpolarization with the same sample and experiment, again confirming that the reported values are representative of individual ELP chains.

Second, in the HyperW experiments, the signal enhancements (i.e., the ratio of peak amplitudes in the hyperpolarized and thermal reference spectra) were approximately 50- to 100-fold for all residues (Figure 3e). This means that, in terms of SNR, the hyperpolarized spectrum corresponded to a >40 h-long conventional experiment (corresponding to *t*_exp_·SNR^2^). The individual SNRs obtained for the hyperpolarized and reference cases are also reported in Figure 3f,g.

The hyperpolarized resonance line shapes resembled those of the reference high-resolution spectrum. In other words, no resolution penalty (as in earlier studies^19,21,26^ ) was introduced, and the dDNP spectrum achieved the same quality as the conventional NMR spectrum but with substantially boosted sensitivity. This convergence was possible due to the above mentioned HySSS.v2 and the tailored apodization function described in the Supporting Information (cf. Figures S4–S7).

Note that the signal enhancement in Figure 3e is not entirely homogeneous. This is due to the fact that below the LCST, propensities for β-turns already exist,^64^ binding all non-Gly residues through transient hydrogen bonding. As a result, the Gly-enhancement is already to some degree higher.

### Above the LCST

The ELP forms condensed coacervate phases, and the HyperW spectra undergo a strong transformation. Under these conditions, the transferred polarization selectively illuminates those residues that remain in dynamic exchange with the surrounding aqueous environment, i.e., those located at the surface of the coacervate particles, while the core residues become masked. The resulting spectra showed several striking features.

First, the surface signals are predominantly enhanced for glycine residues (Figure 3h), while alanine and valine, which contribute to hydrophobic core stabilization within the coacervate,^64^ are only very weakly detectable. This residue-specific signal retention reveals that glycines form the dominant motif at the coacervate-water interface, whereas other residues communicate less efficiently with the solvent. The data thus demonstrate that HyperW-NMR, by virtue of its selective enhancement of solvent-accessible regions, permits a direct and residue-resolved view of the surface composition of phase-separated biomolecular assemblies.

The resulting spectral signatures are characterized by severe line broadening (Figure 3i–k). The line widths increased by a factor of >2 compared to the experiments performed below the LCST. This finding indicates that the HyperW approach indeed strongly boosted broadened resonances that remain below the detection threshold in conventional NMR experiments.

We could not record a thermal equilibrium reference spectrum with the DDNP, as the resonances were too weak for detection in the strongly deuterated dDNP buffer sample (in contrast to Figure 2, where data were recorded under conventional NMR-optimized conditions as indicated in the figure captions and detailed in the Supporting Information). As a result, no enhancements could be calculated. Instead, Figure 3l shows the SNR for the hyperpolarized spectrum, which also represents the key result of the herein reported experiments. The panel shows how the SNR remains weak for all nonglycine residues (black bars) but reaches values up to 100 for glycines (blue bars). In the absence of a reference spectrum, the SNR can be considered as a lower boundary of the signal enhancement, i.e., the inequivalence ε > SNR holds.

In sum, our data show that the surface of ELP coacervates is enriched in glycine residues, most likely as β-turns form, which expose the G3-site to the edges of the ELP condensates (see Figure S8).^60,64^ Thus, the glycine amides also become preferentially solvent exposed, while adjacent and buried residues remain shielded and/or subject to hydrogen bonds that hinder proton exchange with the solvent.

Glycine’s lack of a side chain facilitates hydrophilicity and supports a surface architecture that remains hydrated even in the condensed state. Our data now suggest that glycine-rich surface structures promote reversible interactions with the aqueous environment and mediate phase behavior in response to temperature and pH—a finding well in line with simulation results that led to comparable observations.^64^

Functionally, the glycine-rich surface plays a vital role in mediating the reversible formation^75^ and dissolution of ELP coacervates, an essential feature exploited in applications ranging from synthetic cells to controlled drug delivery systems, where encapsulation efficiency and release kinetics are closely tied to interfacial properties. Furthermore, knowledge of surface composition is critical for the rational design of ELP-based materials with tailored mechanics and responsiveness, including stimuli-adaptive hydrogels and scaffolds for tissue engineering.^70,76^ By providing residue-level resolution of the coacervate–solvent interface, our study lays a mechanistic foundation for decoding ELP function and engineering biomaterials with programmable interfacial behavior.

However, several limitations must also be acknowledged. First, the method relies on the presence of exchangeable protons for polarization transfer, meaning that residues lacking labile hydrogens or those engaged in stable hydrogen bonding networks may be underrepresented or entirely invisible in the spectra. Second, the spatial resolution is fundamentally limited by the diffusive nature of proton exchange, which, while surface-biased, may blur sharp boundaries between interface and bulk under certain dynamic conditions. Finally, while the observed selectivity for glycine residues is informative, it remains challenging to distinguish whether this reflects true compositional enrichment at the surface, differential exchange kinetics, or a combination of both. Indeed, the peaks in Figure 3h are likely a superposition of the surface signals and the resonances of the residual monomers in solution. However, the broad line shapes clearly show that the main contribution does not stem from monomeric species that lead to narrow resonances (cf. Figure 2).

To disentangle the effects of differential exchange kinetics and surface positioning, we conducted a series of experiments with varying interscan delays where polarization is flowing from HyperW to ELP for varying periods. These experiments showed that the glycine residues pick up hyperpolarization quicker from the HyperW than valine or alanine (Supporting Information, Figure S9). Together with HN-exchange rate measurements (Supporting Information, Figure S10), which show that in the bulk of the ELP aggregates, exchange rates are similar among all residues (*k*_ex_ ∼ 15 s^–1^), this finding confirms that the ELP condensate surface is enriched in glycine residues. Indeed, given the homogeneous proton exchange rates in the condensate core, the differential (and *d*1-dependent) enhancement of glycine observed in HyperW cannot be attributed to varying residue-specific exchange rates. Instead, surface localization is required to explain our observations.

In addition, we compared line widths in the HyperW experiments to assess the exposure of the Glycine residues. We found 50 Hz for the Ala/Val residue and ∼60–70 Hz for the Gly residue above the LCST. Nonexchange contributions to transverse relaxation (dipolar relaxation, field inhomogeneity, etc.) account for ∼40 Hz, as judged from spectra recorded below the LCST. Subtracting this baseline leaves an exchange-induced broadening of ∼10 Hz and ∼20–30 Hz, respectively. Using the relation Δυ_ex_ ≈ *k*_ex_/π, these correspond to approximate exchange rates of ∼30 s^–1^ for the Ala/Val residue and ∼60–95 s^–1^ for the Gly residue. Contrasting the homogeneous bulk rates (Figure S10), these differential rates confirm the solvent exposure of the detected glycines.

Note further that the neighboring residues of the surface-exposed glycines are either X or V-type resonances, and one might expect that these neighboring sites receive a similar amount of hyperpolarization as the glycine residues. However, given the typical β-turn motif found in ELP^60,64^ where only the glycine-based amide is not involved in hydrogen-bond formation (Figure S8), a preferential glycine enhancement can be expected.

Yet, it cannot be excluded that the observed weak Ala and Val resonances (Figure 3h) stem from residues adjacent to solvent-exposed glycines, i.e., localized in the same pentapeptide repeat. On the contrary, this is likely, given that we estimate an exchange rate of 30 s^–1^ for these sites, in contrast to the measured 15 s^–1^ for the entire ensemble average.

Furthermore, it is important to note that the presented method does not solve the general problem of signal broadening in large rigid proteins or complexes but addresses sensitivity constraints resulting from the sparseness of surface moieties.

## Conclusion

In this work, we introduce liquid-state surface-enhanced spectroscopy as a novel NMR-based approach to resolve the molecular architecture of soft-matter interfaces with residue-level resolution. By combining hyperpolarized water with surface-selective polarization transfer, we demonstrate that this method enables direct observation of the interfacial composition of phase-separated biomolecular assemblies, such as ELP coacervates. The technique reveals surface-specific residue signatures that are invisible to conventional NMR approaches due to fundamental limitations in sensitivity.

This methodological development represents a conceptual and technical innovation inspired by ideas from surface-enhanced solid-state NMR,^44,45,77–81^ which has significantly advanced our understanding of materials interfaces. Here, we take a first step toward a liquid-state hyperpolarization method to access residue-resolved access to the dynamic and hydrated surfaces of mesoscopic, phase-separated systems in solution.

Importantly, our findings provide a unique structural window into ELP assemblies, whose conformational properties and supramolecular organization remain largely enigmatic despite their widespread use in materials science, biotechnology, and synthetic biology. The ability to pinpoint which residues remain solvent-exposed at the coacervate interface offers crucial insight into the molecular logic underlying ELP phase behavior, surface hydration, and functional presentation.

Our approach thus provides a tool for helping to dissect the interfacial chemistry of complex soft-matter systems with high sensitivity. Given the versatility of NMR detection sequences, the development of a generalizable toolbox based on the herein-proposed proof-of-concept becomes conceivable. Future applications of HyperW to more compact protein assemblies with defined surfaces (e.g., globular proteins and complexes) will further test and expand the scope of this methodology.

## Supplementary Material



## Notes

Experimental details; LCST measurements; DLS data; details on the HySSS.v2; details on the used apodization function; HyperW decay; dDNP experiments with varying *d*1; and proton exchange rates (PDF)
