# Supplementary material for: Residue-Resolved Liquid-State Hyperpolarized NMR of Peptide Condensate Surfaces
Source: J Am Chem Soc. 2025 Sep 25;147(40):36920–7. doi: 10.1021/jacs.5c15053 (PMC12512184; doi:10.1021/jacs.5c15053)
Supplement: Supplementary file 1 [file ja5c15053_si_001.pdf]

Residue-Resolved Liquid-State Hyperpolarized NMR of Peptide Condensate Surfaces

Dörte Brandis[1,2], Ertan Turhan[1], Milan Zachrdla[1], Dennis Kurzbach[1,]*
[1]Institute of Biological Chemistry, Faculty of Chemistry, University of Vienna, Währinger Str. 38, 1090 Vienna, Austria
[2]University of Vienna, Vienna Doctoral School in Chemistry (DoSChem), Währinger Str. 42, 1090 Vienna, Austria
**E-Mail: dennis.kurzbach@univie.ac.at*

Supporting information

Table of Contents

Experimental Details Page S1

Figure S1. OD Measurements Page S4

Figure S2. DLS Measurements Page S5

Figure S3. HyperW Decay Page S5

Figure S4. HySSS Page S7

Figure S5. HySSS interior Page S9

Figure S6. Raw Hyperpolarized Data Page S9

Figure S7. Apodization Functions Page S10

Figure S8. Beta-Turn Page S11

Figure S9. Experiments With Varying d1 Page S11

Figure S10. Bulk Exchange Rates Page S12

Experimental

Sample Production: ELPs were prepared as described in our earlier work.[1]

The entire ELP sequence was:

VGVPGVGVPGGGVPGAGVPGVGVPGVGVPGVGVPGGGVPGAGVPGGGVPGVGVPGVGVPGGGVPGAGVPGV
GVPGVGVPGVGVPGGGVPGAGVPGGGVPGVGVPGVGVPGGGVPGAGVPGVGVPGVGVPGVGVPGGGVPGAG
VPGGGVPGVGVPGVGVPGGGVPGAGVPGVGVPGVGVPGVGVPGGGVPGAGVPGGGVPGVGVPGVGVPGGGV
PGAGVPGVGVPGVGVPGVGVPGGGVPGAGVPGGGVPGVGVPGVGVPGGGVPGAGVPGVGVPGVGVPGVGVP
GGGVPGAGVPGGGVPGVGVPGVGVPGGGVPGAGVPGVGVPGVGVPGVGVPGGGVPGAGVPGGGVPGVGVPG
VGVPGGGVPGAGVPGVGVPGVGVPGVGVPGGGVPGAGVPGGGVPGVGVPGVGVPGGGVPGAGVPGVGVPGV
GVPGVGVPGGGVPGAGVPGGGVPGENLYFQ

Importantly, for thermal equilibrium measurements (Fig. 2 of the main text), the sample was prepared in 10% D_2O, pH 6.5, and at a concentration of 2 mg/mL. These conditions were chosen to optimize amide proton visibility in standard liquid-state NMR. This composition exhibits sensitivity in conventional spectra governed by Boltzmann polarization, resulting in slow exchange at reduced pH and high protonation levels.

In contrast, HyperW-enhanced NMR requires a fundamentally different optimization strategy. Here, the sample consisted of (after DNP, dissolution, and mixing) 96% D_2O at pH 7.0 and a concentration of 0.6 mg/mL, reflecting the unique requirements of HyperW experiments for efficient polarization transfer via proton exchange. The high D_2O content minimizes water relaxation during hyperpolarization transfer, thereby maximizing signal enhancement throughout the entire course of the NMR detection. A slightly increased pH accelerates exchange rates of labile protons, particularly the backbone amide hydrogens, improving polarization transfer efficiency. The lower concentration is imposed by instrumental constraints related to sample delivery and mixing dynamics in the HyperW protocol.

While the two conditions differ in composition, they are tailored to their respective detection regimes: the thermal setup maximizes steady-state resolution, whereas the HyperW setup maximizes transient signal amplification. Importantly, however, the different sample compositions cause visible peaks in the spectra above the LCST in Fig. 2 (thermal equilibrium experiments) of the main text, in contrast to their absence in Fig. 3 of the main text (HyperW experiments).

Hyperpolarized NMR: For DNP 200 mL of a solution of 30 mM TEMPOL in a mixture of 15% glycerol-d_8 and 85% H_2O were hyperpolarized at a temperature of 1.4 K in a magnetic field of 6.7 T for ~ 5400 s using continuous-wave microwave irradiation at 188 GHz. DNP samples were always freshly prepared to avoid ripening effects.[2] A VDI microwave source was used together with a 16x frequency multiplier that provided an output power for the microwave of ca. 50 mW. The magnet-cryostat combination was purchased from Cryogenic Ltd. and operated as described in reference [3].

For the detection of the proton solid-state polarization, a 400 MHz Bruker NEO system was adapted to a ^1H resonance frequency of 285.3 MHz. The detection circuit and the external tune-and-match system were home-built, as described in reference [4]. To monitor the build-up, detection pulses with a flip angle of 1 degree were applied every 5 s.

After DNP, the sample was dissolved with a burst of 5 mL D_2O at 1.5 MPa as described in reference [3]. The hyperpolarized liquid was then pushed with helium gas at 0.7 MPa to the HySSS.v2. The dissolution process employed a home-built pressure heater actuated with an Arduino microcontroller. A predefined hyperpolarized solution (350 µL) was injected into a Shigemi NMR tube (Shigemi Ltd.) using the HySSS.v2 prototype described below with a second Arduino microcontroller within 1.7 s. [5]

This experimental setup led to 4% protonation in the hyperpolarized sample, which has been previously shown to provide the maximum product of polarization and relaxation time, $P(^1H)\cdot T_{1,H2O}$.[6] This product is responsible for the overall signal enhancement in HyperW applications.

Detection in the liquid state was carried out using a 700 MHz Bruker HDIII spectrometer equipped with a QCIF helium-cooled cryogenic probe. The pulse sequence for detection corresponded to a BEST-HMQC [7-9]. We used PC9 and RSNOB[10] selective 90° and 180° pulses covering a bandwidth of 4 ppm centered around a carrier frequency of 9.0 ppm to excite and invert the protons (not a 120° pulse as the polarization recovery mechanism is different from thermal equilibrium NMR). The pulse lengths were 2.06 ms and 686 µsec, respectively. The 90° pulse for the ^{15}N channel was 37 µsec long. The ^{15}N carrier frequency was adjusted to 117 ppm. Heteronuclear decoupling was achieved using the GARP[11] scheme as preinstalled in Bruker TOPSPIN 4. The recycling delay 2xd1 was set to 0.15 s, the acquisition time was 0.1 s, leading to a total recycling time of 0.31 s. In total, 256 t_1 increments were recorded with a step length of 0.2 ms.

The protein solutions for dDNP contained 2 mg/mL of ELP before three-fold dilution with hyperpolarized water, as described in the main text. After dilution, the sample volume was 450 mL, and the peptide concentration was consequently 2/3 mg/mL.

Thermal equilibrium reference spectra were recorded using the same pulse sequences but with 512 t_1-increments instead of 256 and 256 averages per FID instead of 2 (sample composition either as in the dDNP experiments to determine the enhancement, or, as outlined above to optimize thermal magnetization).

Data Processing: All data were processed with Bruker TopSpin 4 and home-written scripts in MATLAB. All data were zero-filled to 4 times the original FID length, and the baseline was corrected using 5th-order polynomials after the Fourier Transform. For conventional NMR experiments, a 60° shift q-sine bell was used as the apodization function. In contrast, for the DDNP experiments, the apodization function in the t_1 dimension was multiplied by the inverse exponential decay of the hyperpolarized water $1/(\exp(-R_{1,\mathrm{HyperW}}t))$ with $R_{1,\mathrm{HyperW}}$ being the efficient decay of the water hyperpolarization.[12]

In brief, the conventional squared 60° shifted q-sine bell function (q=3) is defined as:

$$\omega_{sine}(n) = \sin\left(\frac{n\pi}{N} + \vartheta\right)^3 \quad (1)$$

Where $\vartheta = 60°$and n is the t_1 increment index (ranging from 0 to N-1), and N is the total number of t_1 points.

To account for the decay of hyperpolarized water, we use the known effective HyperW relaxation time $T_{1,\mathrm{eff,HyperW}}$, (see Fig. S3 for the determination) and define the decay during t_1 incrementation as:

$$d(n) = \exp\left(-\frac{n\Delta t_1}{T_{1,eff,\mathrm{HyperW}}}\right) \qquad (2)$$

Where Δt_1 corresponded to d1+aq (i.e., not the evolution time increment, but the time between scans). The final apodization window applied to each t_1 increment is then:

$$\omega(n) = \omega_{sine}(n)/d(n) \quad (3)$$

This formulation corrects for the influence of the HyperW decay during t_1 incrementation. This decay, in fact, causes each increment in a HyperW-based 2D experiment to be recorded with a different starting polarization, such that the indirect FID decays with two exponential functions with rates $R_{1,\mathrm{HyperW}}$ and $R_{1,\mathrm{intrinisic}}$, *i.e.*, the intrinsic FID decay rate constant (i.e., without any hyperpolarization) plus the decay rate constant of the hyperpolarized water. The factor $d(n)$, thus, effectively cancels the contribution from the water to the FID, leaving only the intrinsic contributions to the linewidth (Fig. S7). The corrected result is shown in the main text Fig. 3, and the hyperpolarized spectra with the simple sine bell apodization in Fig. S6, where the effectiveness of this correction is demonstrated on both raw and processed data.

In practice, the function d(n) can also be obtained by the ratios of the exponential envelopes of the FIDs along t_1 in the hyperpolarized and thermal equilibrium reference spectra. Importantly, d(n) needs to be normalized such that $\int_{t=0}^{t\to\infty} d(n)\, dt = 1$ to not change the resonance integral.

Lorentzian fitting

To quantitatively assess the effects of hyperpolarization and phase separation on the spectral properties of ELP residues, individual cross-sections through selected peaks in the 2D ^{1}H–^{15}N spectra were analyzed using Lorentzian line shape fitting. This approach enables the extraction of peak linewidths (Γ) and amplitudes, which serve as proxies for molecular mobility and spectral resolution, respectively.

The fits were performed using nonlinear least-squares minimization in MATLAB. Each selected peak was modeled as a single Lorentzian function of the form:

$$I(\nu) = \frac{A}{\pi} \frac{\Gamma/2}{(\nu - \nu_0)^2 + (\Gamma/2)^2} \qquad (4)$$

where $I(\nu)$ is the signal intensity at frequency ν, A is the area under the peak (proportional to signal strength), ν_0 is the peak center, and Γ is the full width at half maximum (FWHM).

Fitting was performed over a ±0.1 ppm window centered on the peak maximum to ensure exclusion of overlapping signals or baseline distortions. The extracted linewidths and peak intensities were then compared across temperature conditions and between hyperpolarized and thermal reference spectra. Importantly, consistency in linewidths between thermal and HyperW spectra below the LCST validated the reliability of the fit and confirmed that hyperpolarization did not distort intrinsic line shapes. Above the LCST, broadened linewidths in glycine peaks indicated decreased mobility at the coacervate surface, further supporting their interfacial localization.

All fits achieved R^2 values >0.98, and residuals showed no systematic deviations, indicating excellent model agreement.

DLS Measurements

DLS data have been recorded at an in-house-built DLS setup, consisting of a focused laser beam (λ = 528 nm) and a single photon avalanche detector connected to a buffered counter (using a NI PCI-6601). Scattered photons were recorded at an angle of 90°. Measurements were performed at a temperature of 35 °C, with a time step of 30µs and a total length of 150 s. The intensity autocorrelation function (ACF) was calculated for each of these measurements. For lag times larger than 1 ms, a binning procedure was used to smooth the ACF. ACF data were analyzed using the CONTIN algorithm.[13-15]

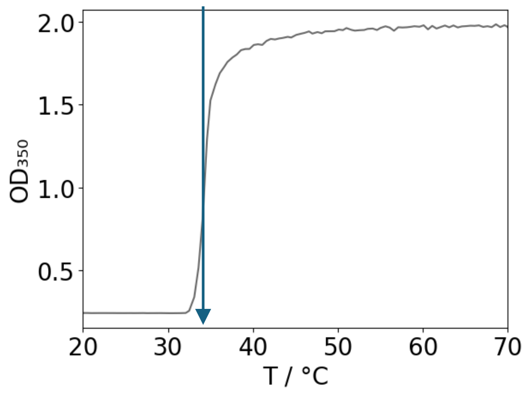

Figure S1. Optical density of the used ELP under the same conditions as the NMR measurements samples at a wavelength of 350 nm is dependent on the sample temperature. A temperature of 35° C to the best compromise between NMR detection and sample temperature above the LCST, which is <34° C (marked by the arrow in the Figure).

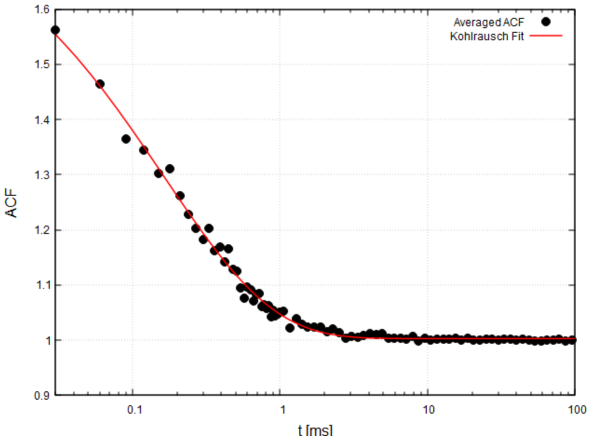

Figure S2: DLS data: Intensity autocorrelation function (ACF) and fit used to obtain the size distribution of ELP at 35 °C. The data was fitted to a hydrodynamic radius of 156 nm.

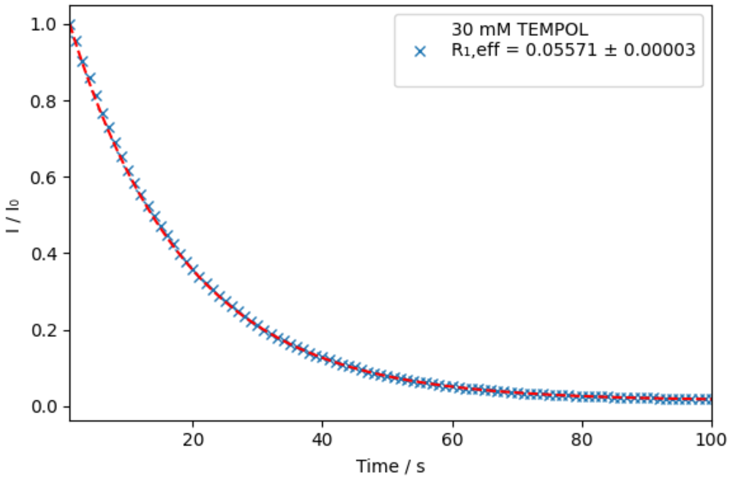

Figure S3. Decay of water hyperpolarization. HyperW was contributing to the signal intensity for ca. 1 min. The effective R_1 rate was fitted to 0.06 s^{-1} +/- 0.01 s^{-1}.

Estimation of ELP Aggregate Molecular Weight

To estimate the approximate molecular weight of the ELP coacervate globules studied herein, we employed a polymer-physics-based rule-of-thumb grounded in Flory–Huggins theory for swollen globules in dilute aqueous environments. Taking the experimentally determined hydrodynamic radius (R_h) of 156 nm, we considered the coacervate as a spherical globule composed of collapsed ELP chains in a good solvent regime with a polymer volume fraction ϕ = 0.02, a value supported by literature for highly hydrated protein assemblies[16].

The total volume of a single globule was calculated as:

$$V_{globule} = \frac{4}{3}\pi R_h^3 = 1.59 \cdot 10^7 nm^3$$

With a volume fraction of ϕ=0.02 the ELP material occupies approximately:

$$V_{ELP} = \phi V_{globule} = 3.18 \cdot 10^5 nm^3$$

Assuming a typical hydrated volume of 60 nm^3 per ELP chain (corresponding to 450 residues at ~0.135 nm^3 per residue[17]), this implies approximately:

$$N_{Chains} = \phi V_{globule}/60\ nm^3 \approx 5300$$

Given the average molecular weight MW of a single chain is ~49.5 kDa, the total molecular weight of the globule can be estimated as:

$$M_{total} = N_{Chains} MW \approx 262\ MDa$$

This estimate is based on assumptions from accepted polymer theory. However, the precise molecular content may vary due to sequence-specific compaction, hydration dynamics, and ionic strength. Nonetheless, even when variations of two orders of magnitude are considered, this value clearly shows that the peptide aggregates feature more than 1 MDa in cumulative molecular weight.

The Hybrid Sample Shuttling System (HySSS).

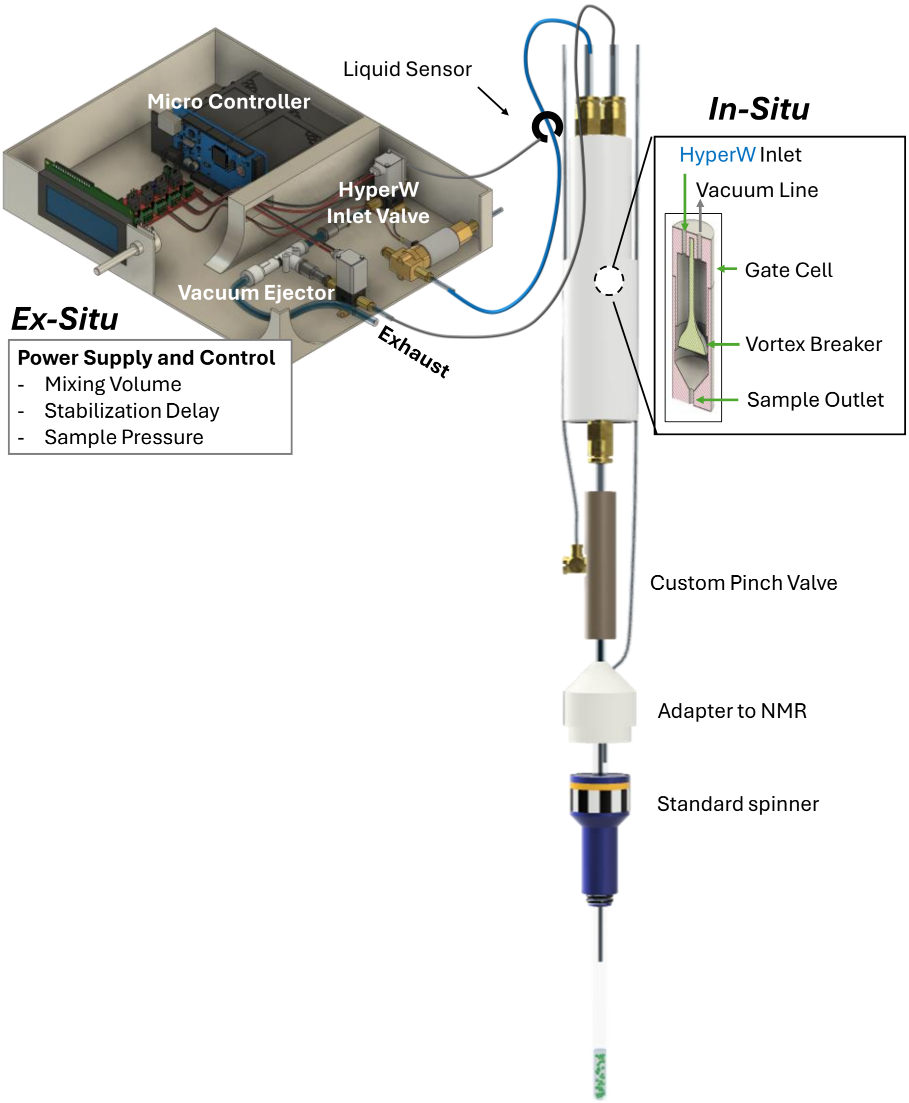

Figure S4. HySSS.v2 design. The gate cell fits *in-situ* into the bore of a standard NMR spectrometer. It can be connected to a standard NMR spinner and tube. The control system is located *ex-situ*, outside of the bore. It guides HyperW and connects to a vacuum line. A liquid sensor detects the time when the HyperW arrives from the DNP system to start the strictly timed sample preparation phase.

The first implementation of a HySSS was described in reference [18]. This system was mounted on the top of the NMR spectrometer and did not provide any control over sample temperature or pressure. However, to handle ELP aggregates a stable temperature needed to be maintained during mixing of the ELP suspension with the HyperW. We needed to make sure that the temperature remained stably above/below the LCST in our different experiments. Therefore, the HySSS had to be updated, such that the chamber (gate cell) for mixing and sample degassing moved directly above the NMR tube into the bore of the NMR spectrometer, such that it could be tempered in the exact same way as the NMR sample tube to the same target temperatures. The design that made this possible is shown in Fig. S4.

The centerpiece of this instrument is a “gate cell” that receives HyperW arriving from the dDNP system and provides a volume for mixing. The gate cell featured an aspect ratio to fit directly into the bore of a standard NMR spectrometer. Equipped with a specially designed “vortex-breaker”[18], it could homogenize and degas

the solution within milliseconds. The vortex breaker is a hydrodynamics element that enables a vacuum (or pressure) to be evenly applied across the HyperW reservoir in the volume provided at the bottom of the gate cell, ensuring minimal sample movement.

The gate cell's outlet towards the NMR tube is controlled by a custom-made pinch valve (PV) that fits within the narrow bore of a standard NMR spectrometer and is actuated *via* an external pressure supply. This design ensures that the entire HySSS.v2 can be placed directly above the NMR tubes so that samples always remain in a homogenous magnetic field and at a controlled temperature throughout the sample preparation phase. Furthermore, it was connected to a vacuum line in order to control the pressure within the mixing chamber.

The injection process of the HyperW is triggered through the optical sensor upon detecting the liquid traveling to the HySSS.v2. Upon detection a strictly timed process is carried out leading in injection of precise amounts of hyperpolarized liquid in to the NMR tube. The entirety from dissolution to injection, depending on the preset parameters is completed within 1.7 s. The following sequence of events is then carried out:

a) Collecting: Detected HyperW gets collected in the gate cell for 500 ms. The remaining liquid is diverted into waste.
b) Degassing: For 1000 ms the collected liquid gets degassed to ensures removal of gas inclusions/bubbles
c) Injecting: The custom pinch valve forwards a precise volume into the NMR tube predetermined by the chosen delay for the PV opening. (error <50 µL).

The function schematic of the injection into the NMR tube is shown in Fig. S5, carried out once the optical sensor (red square) detects passing liquid. Upon passing the optical sensor, the liquid enters the gate cell. Once the gate cell is filled (volume below the vortex breaker piston), the three-way valve (HypV) changes position and forwards the remaining liquid into the waste. At the same time, this prevents the chase gas from interfering with the subsequent degassing process inside the gate cell, for which the vacuum line is opened. Subsequent to degassing, the two two-way valves (D1 and D2), responsible for degassing are closed again, and then both the pinch valve (brown) and the three-way valve (HypV) are reopened. This allows the chase gas to push parts of the liquid present in the gate cell into the NMR tube. The injected amount can be controlled by changing the delay, which determines the time the pinch valve remains open. Furthermore, collection time and degassing time can be adjusted to the specific need, thus allowing the system to be transferred rapidly to new settings and different distances while maintaining reproducibility.

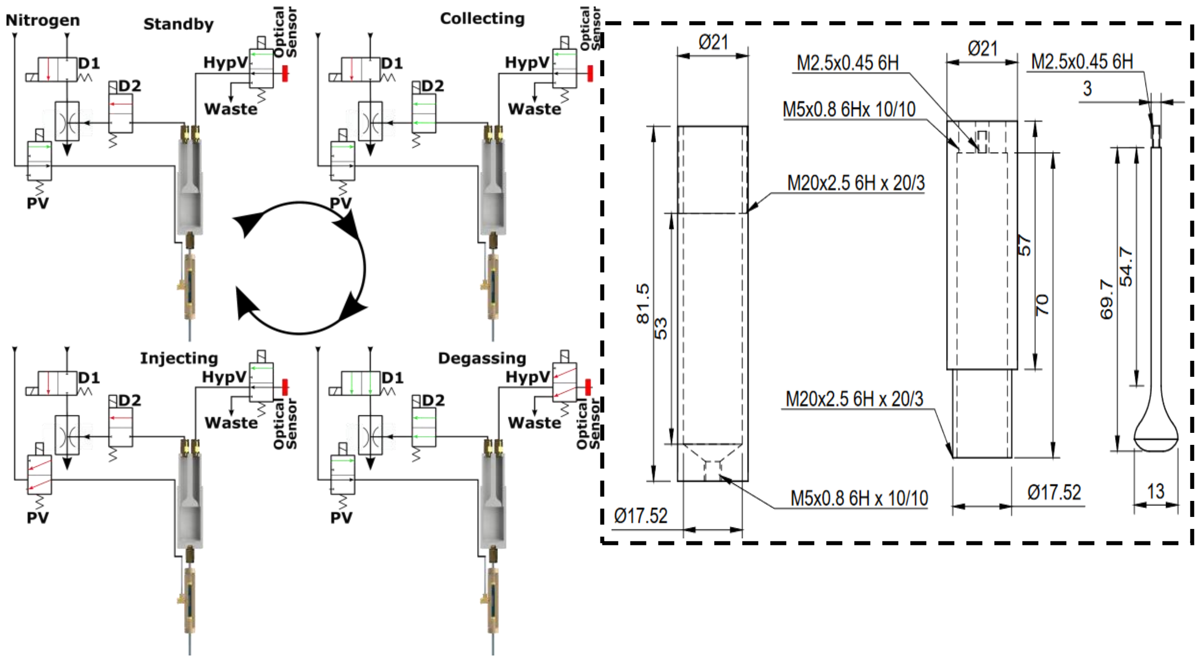

Figure S5. Representation of the utilized components. Left: Vales and liquid flow controlling the HySSS throughout an injection cycle. Right: Dimensions of the gate cell. Those were chosen accordingly to ensure fit into Bruker narrow bore spectrometers.

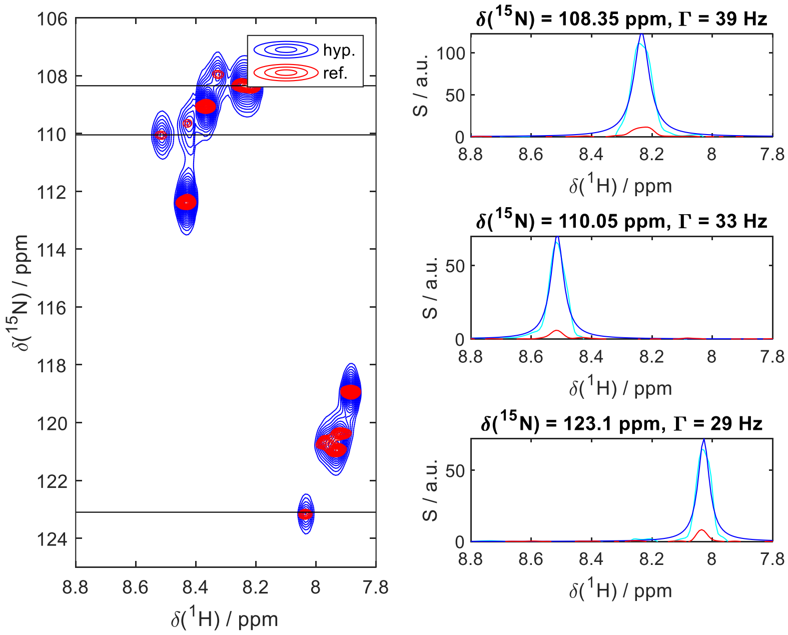

Figure S6. Hyperpolarized spectrum (blue) stemming from the same experiment as the data in Fig. 3a of the main text, but apodized with a conventional shifted sine bell window. The red spectrum is the same thermal equilibrium reference as in the main text. When the window is multiplied by the inverse exponential decay of the hyperpolarized water (Fig. S3), then the spectrum recovers its undistorted lineshape in the indirect ^{15}N dimension. Note how the line shape in the direct ^{1}H dimension remains unchanged.

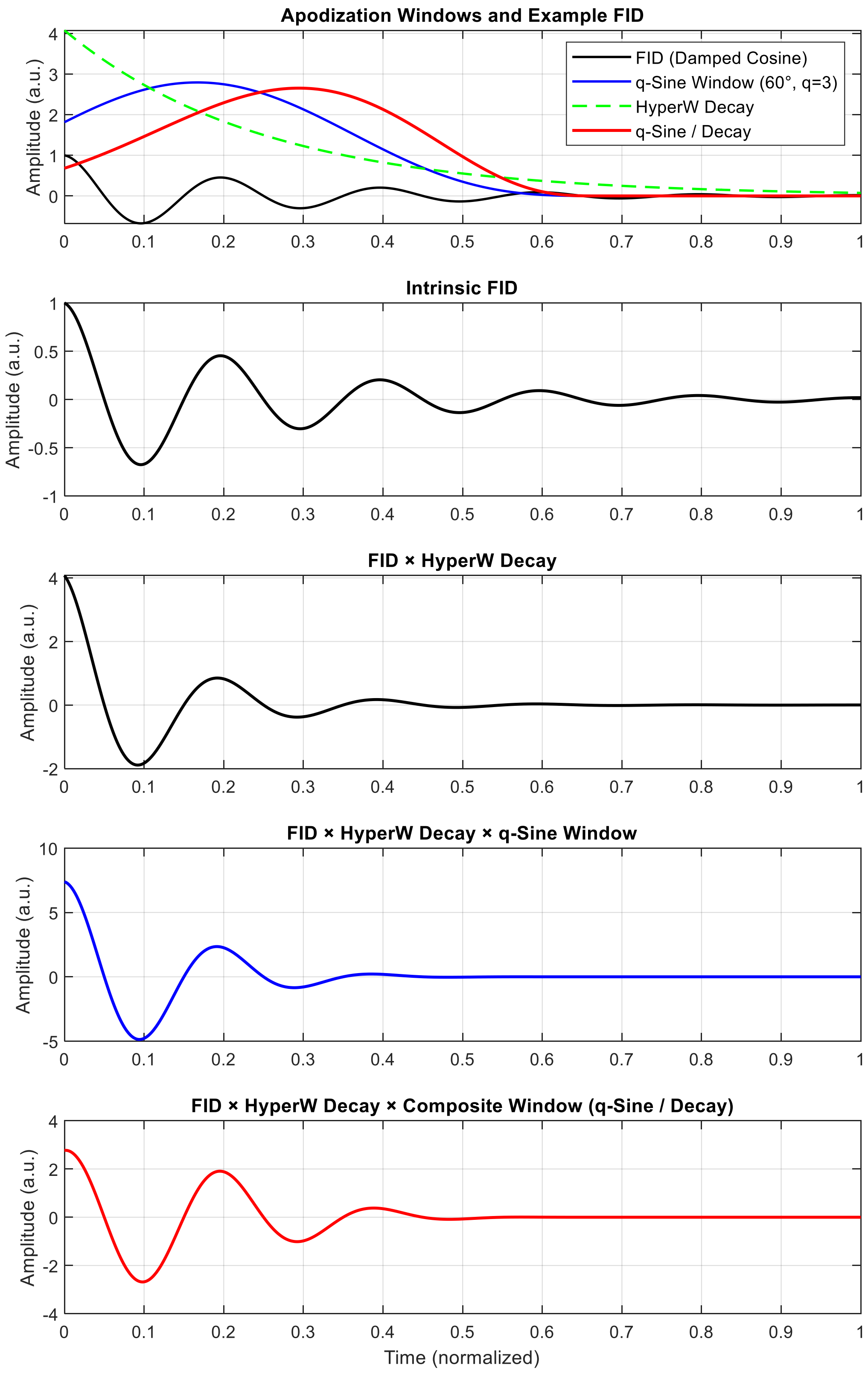
Apodization Windows and Example FID
FID (Damped Cosine)
q-Sine Window (60°, q=3)
HyperW Decay
q-Sine / Decay
Intrinsic FID
FID × HyperW Decay
FID × HyperW Decay × q-Sine Window
FID × HyperW Decay × Composite Window (q-Sine / Decay)
Amplitude (a.u.)
Time (normalized)

Figure S7. Visualization of the HyperW-tailored apodization function along the indirect recorded dimension. Panel 1 shows the original simulated FID (black), along with three normalized apodization functions: a 60°-shifted q-sine window (blue), the exponential HyperW decay function (green), and a composite apodization window constructed by dividing the q-sine by the decay (red). Panels 2–5 show the successive application of each function to the FID: (2) the raw damped cosine FID, (3) the signal after exponential decay simulating spin relaxation, (4) the decayed signal apodized with the q-sine window, and (5) the decayed signal apodized with the composite window. The composite apodization recovers the intrinsic FID shape after apodization, while the conventional window function retains overly intense points for short times.

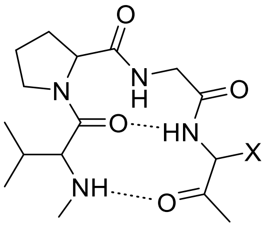

Figure S8. Typical β-turn found at the edges of aggregated ELPs. The Gly-amide proton is not involved in hydrogen bonding, while the neighboring Val and X-residues are. This explains why glycines become preferentially hyperpolarized at the surface, and neighboring residues only to a lesser extent.

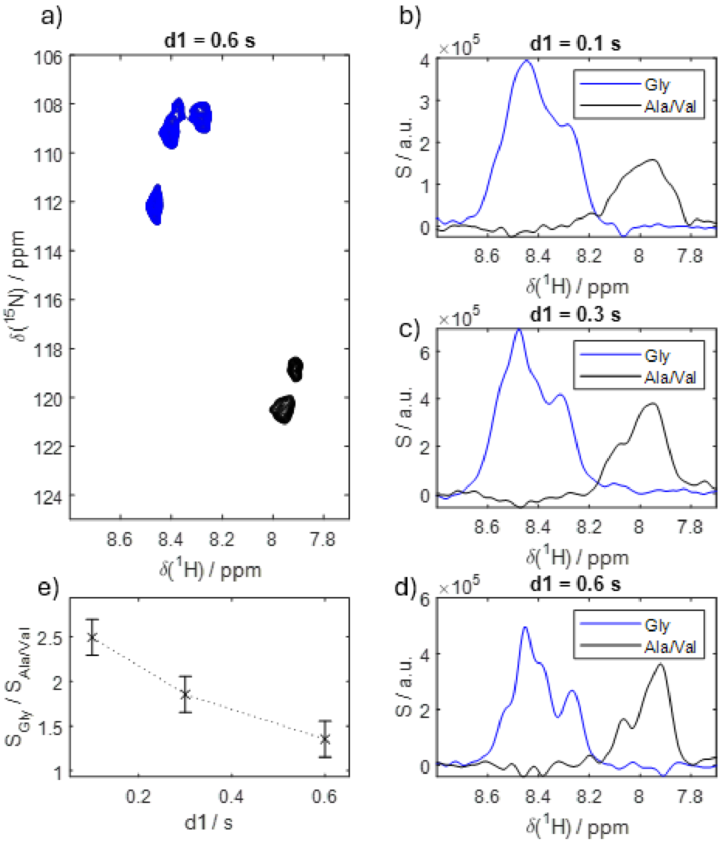

Figure S9. a) Exemplary HyperW-enhanced spectrum above the LCST with a d1 of 0.6 s (as opposed to 0.3 in the original manuscript). b-d) Sum projections of the glycine (blue) and alanine/valine (black) signals. With increasing d1, the contribution of the Ala/Val residues increases and vice versa. e) Ratio of signal intensities between glycine and alanine/valine resonances. Evidently, shorter d1 leads to stronger enhancement of the Gly residues. These finding highlights that the glycine residues indeed pick up hyperpolarization faster from the solvent than Ala/Val residues.

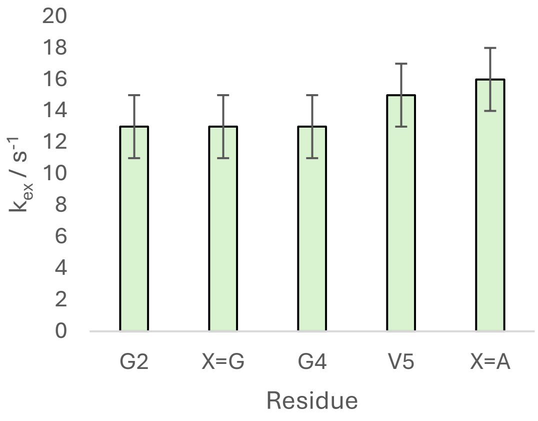

Figure S10. HN amide exchange rates determined with ^{13}C-direct detected experiments as detailed in reference [1]. These rates reflect the average proton exchange over all residues (bulk and surface).

References

1. Brandis, D. r.; Kadeřávek, P.; Kurzbach, D., The Internal Structural Dynamics of Elastin-Like Polypeptide Assemblies by 13C-Direct Detected NMR Spectroscopy. *Anal.Chem.* **2025,** *97* (7), 3937-3944.
2. Weber, E. M. M.; Sicoli, G.; Vezin, H.; Frébourg, G.; Abergel, D.; Bodenhausen, G.; Kurzbach, D., Sample Ripening through Nanophase Separation Influences the Performance of Dynamic Nuclear Polarization. *Angew. Chem. Int. Ed.* **2018**, 10.1002/anie.201800493.
3. Kress, T.; Che, K.; Epasto, L. M.; Kozak, F.; Negroni, M.; Olsen, G. L.; Selimovic, A.; Kurzbach, D., A novel sample handling system for dissolution dynamic nuclear polarization experiments. *Magnetic Resonance* **2021,** *2*, 387-394.
4. Baudin, M.; Vuichoud, B.; Bornet, A.; Milani, J.; Bodenhausen, G.; jannin, S., A Cryogen-Free 9.4 T System for Dynamic Nuclear Polarization. *under review.* **2018**.
5. Turhan, E.; Pötzl, C.; Keil, W.; Negroni, M.; Kouřil, K.; Meier, B.; Romero, J. A.; Kazimierczuk, K.; Goldberga, I.; Azaïs, T.; Kurzbach, D., Biphasic NMR of Hyperpolarized Suspensions–Real-Time Monitoring of Solute-to-Solid Conversion to Watch Materials Grow. *The Journal of Physical Chemistry C* **2023,** *127* (39), 19591-19598.
6. Hilty, C.; Kurzbach, D.; Frydman, L., Hyperpolarized water as universal sensitivity booster in biomolecular NMR. *Nat Protoc* **2022,** *17* (7), 1621-1657.
7. Schanda, P.; Forge, V.; Brutscher, B., Protein folding and unfolding studied at atomic resolution by fast two-dimensional NMR spectroscopy. *Proceedings of the National Academy of Sciences of the United States of America* **2007,** *104* (27), 11257-11262.
8. Schanda, P.; Kupce, E.; Brutscher, B., SOFAST-HMQC experiments for recording two-dimensional heteronuclear correlation spectra of proteins within a few seconds. *J Biomol NMR* **2005,** *33* (4), 199-211.
9. Schanda, P.; Brutscher, B., Very Fast Two-Dimensional NMR Spectroscopy for Real-Time Investigation of Dynamic Events in Proteins on the Time Scale of Seconds. *Journal of the American Chemical Society* **2005,** *127* (22), 8014-8015.

10. Ying, L.; Benjamin, W. J.; Chad, R. M., Selective refocusing pulses in magic-angle spinning NMR: Characterization and applications to multi-dimensional protein spectroscopy. *Journal of Magnetic Resonance* **2006,** *179* (2), 206-216.
11. Shaka, A. J.; Barker, P. B.; Freeman, R., Computer-optimized decoupling scheme for wideband applications and low-level operation. *Journal of Magnetic Resonance (1969)* **1985,** *64* (3), 547-552.
12. Negroni, M.; Kurzbach, D., Residue-resolved monitoring of protein hyperpolarization at sub-second time resolution. *Commun Chem* **2021,** *4* (1), 147.
13. Provencher, S. W., Inverse problems in polymer characterization: direct analysis of polydispersity with photon correlation spectroscopy. *Die Makromolekulare Chemie: Macromolecular Chemistry and Physics* **1979,** *180* (1), 201-209.
14. Provencher, S. W., A constrained regularization method for inverting data represented by linear algebraic or integral equations. *Computer Physics Communications* **1982,** *27* (3), 213-227.
15. Salazar, M.; Srivastav, H.; Srivastava, A.; Srivastava, S., A user-friendly graphical user interface for dynamic light scattering data analysis. *Soft Matter* **2023,** *19* (34), 6535-6544.
16. Rubinstein, M.; Colby, R. H., *Polymer physics*. Oxford university press New York: 2003; Vol. 23.
17. Counterman, A. E.; Clemmer, D. E., Volumes of individual amino acid residues in gas-phase peptide ions. *Journal of the American Chemical Society* **1999,** *121* (16), 4031-4039.
18. Negroni, M.; Turhan, E.; Kress, T.; Ceillier, M.; Jannin, S.; Kurzbach, D., Fremy's Salt as a Low-Persistence Hyperpolarization Agent: Efficient Dynamic Nuclear Polarization Plus Rapid Radical Scavenging. *J Am Chem Soc* **2022,** *144* (45), 20680-20686.
